# Cases of Fracture of the Skull, Presenting Some Singular Phenomena

**Published:** 1840-06

**Authors:** William G. Dickinson

**Affiliations:** Nashville, Tennessee


Art. II.—*Cases of Fracture of the Skull presenting some singular phenomena.* Read before the Medical Society of Tennessee, and reported for the Western Journal. By William G. Dickinson, M. D., of Nashville, Tennessee.

J. D. aged twenty-two years was thrown by a horse while at full speed, and was found lying on his side; his head protected by a felt hat, was about ten inches from a large post. It was *believed* that the head came in contact with the ground and post at the same time, but no external sign indicated that his head had been injured; no puffiness, or the least abrasion of the harry scalp could be detected by the most careful examination.

He was insensible, breathing slowly, but not laboriously:

in this respect there was no change while he lived. On the second, third, fourth and fifth days he swallowed whatever was put in his mouth, his urine and fæces were passed involuntarily, and he died on the tenth day after receiving the injury.

### EXAMINATION TWENTY HOURS AFTER DEATH.

The whole of the hairy scalp was removed, the pericranum adhered to the bone throughout, except where it had been separated by the surgeon.

On raising the skull about two ounces of semi-transparent fluid escaped. The longitudinal and lateral sinuses were filled with dark coagulated blood. The veins of the cerebrum were but partially filled even at their base, while those of the cerebellum were distended almost to bursting, and the whole mass of the cerebellum was enveloped in three or four ounces of semi-purulent fluid slightly tinged with blood.

On raising the dura mater from the base of the skull a fracture was found extending entirely across its base through the petrous portion of the temporal bone, and reaching about one inch above the external ear on each side. On the anterior part of the petrous portion of the temporal bone of the right side was an isolated portion of the bone, but connected with the line of the fracture on one side, about half an inch in width and three-fourths of an inch in length, which was forced from within outward upon the internal ear, as if by a blow of some small instrument impinged on the internal surface of the bone. On the left portion of the pars petrosa was a similar isolated piece also connected with the line of fracture, of about the same width as the former and more than one inch in length, its longest diameter corresponding with the lateral diameter of the skull. No remark was made or conjecture hazarded, by the professional gentlemen present, as to the cause which had produced this novel appearance.

These pieces were detached from the dura mater, which, except a slight effusion externally, where the vessels were torn from the isolated portions of bone, seemed in a perfectly healthy state; as it did also along the whole extent of the fracture; and the brain and other parts adjacent had the like appearance of exemption from the effects of the shock, which produced such fearful effects on the contiguous bony structure.

*Case 2d.*—W. aged about twenty-five years, received a blow from a flat surface on the left side of his head, parallel to the superior orbital process; the force of the blow was expended on the os frontis where it, the temporal and parietal bones join. He was standing, and did not fall, but reeled and staggerd backwards—he died on the third day.

*Autopsy.*—The skin and external integuments were entirely sound; nor was there the least vestige of fracture on the external parietes of the cranium. But on the superior orbital plate of the os frontis, was a fracture which included a piece of the orbital process, one inch in length and three-fourths of an inch broad. The isolated piece was irregular and angular; its longest diameter from left to right. This pressed upon the orbit of the eye from within, as if the blow had been received on the inside of the skull, from above downwards: and the lamellated nature of the fracture showed that it could never have been forced in upon the brain, the sides being scaled off from within outward and lying upon the part from which it was separated, not unlike the squamous suture, the broadest part being external. The portion of the fracture most distant from, and opposite to the point of injury, was perpendicular and nearly straight, constituting as it were the base of the fractured piece. The dura mater over this fractured portion was sound and there was nothing in its appearance to indicate the injury which the bony structure had sustained beneath.

I have purposely passed over the symptoms and treatment; it is sufficient to say that the cases though widely distant, were treated by professional men, eminent for their practical and scientific acquirements. As the symptoms gave no indications of the injury which was found to exist after death, of course the treatment had no reference to it. Doctor R. C. K. Martin was attending surgeon, and by him the examination was made, in the presence of Dr. Felix Robertson, President of the Medical Society of Tennessee, and myself. Both bear testimony to the correctness and fidelity with which the facts are here stated.

I wish simply to call the attention of the profession to the fact, that the arch of the cranium may give way at any point *between* the point of injury and the point of resistance, always supposing the side opposite to the point of injury, to be the point of resistance, and that whenever it does so give way, from the very nature of its form, the parts isolated must of necessity be forced from within outward; and that it is always liable to this kind of injury when the surface is large on which the blow is received, and particularly so if the impinging force is in a degree less than that which would break through the arch at the point where the injury is received. It is also probable that the bones of an old person or a very young one would not suffer in this way; the former would be too brittle, and the latter too soft; and both would give way at the point of injury or not at all. It may be that the shock which the substance of the brain must sustain in receiving an injury of this character may always be fatal, as it was in the cases narrated. Still my object will be attained by calling the attention of the Society to the fact that the skull is liable to this kind of injury.

*Nashville*, May, 1840.

